# Conversational AI agent for precision oncology: AI-HOPE-WNT integrates clinical and genomic data to investigate WNT pathway dysregulation in colorectal cancer

**DOI:** 10.3389/frai.2025.1624797

**Published:** 2025-08-11

**Authors:** Ei-Wen Yang, Brigette Waldrup, Enrique Velazquez-Villarreal

**Affiliations:** ^1^PolyAgent, San Francisco, CA, United States; ^2^Department of Integrative Translational Sciences, Beckman Research Institute of City of Hope, Duarte, CA, United States; ^3^City of Hope Comprehensive Cancer Center, Duarte, CA, United States

**Keywords:** artificial intelligence, AI agent, precision oncology, large language model (LLM), medical AI, healthcare AI, colorectal cancer, WNT pathway

## Abstract

**Introduction:**

The WNT signaling pathway is a key driver of colorectal cancer (CRC) initiation and progression, particularly in early-onset CRC (EOCRC) among underserved populations. However, interrogating WNT pathway dysregulation across clinical and genomic dimensions remains technically challenging, limiting both translational insight and personalized intervention strategies. To address this gap, we developed AI-HOPE-WNT, the first conversational artificial intelligence (AI) agent purpose-built to investigate WNT signaling in CRC using natural language–driven, integrative bioinformatics.

**Methods:**

AI-HOPE-WNT employs a modular architecture combining large language models (LLMs), a natural language-to-code engine, and a backend statistical workflow interfaced with harmonized data from cBioPortal. Unlike general-purpose platforms, AI-HOPE-WNT is uniquely optimized for WNT-specific precision oncology. The tool supports mutation frequency analysis, odds ratio testing, survival modeling, and subgroup stratification by genomic, clinical, and demographic variables. To validate the platform, we recapitulated findings from two previous studies examining WNT pathway alterations in high-risk CRC populations, including mutation prevalence in RNF43 and AXIN2 and survival outcomes associated with WNT pathway status across ethnic and age subgroups. Exploratory queries further assessed treatment response, co-mutation patterns, and population-specific trends.

**Results:**

In recapitulation analyses, AI-HOPE-WNT reproduced key trends from prior work, including improved survival in WNT-altered EOCRC and higher RNF43 mutation rates in Hispanic/Latino (H/L) populations compared to non-Hispanic White (NHW) people. Exploratory analyses revealed several novel findings. Among FOLFOX-treated EOCRC patients, APC mutations were associated with significantly different survival outcomes (*p* = 0.043). RNF43-mutant tumors showed worse survival in metastatic versus primary cases (*p* = 0.028). AXIN1 and APC co-mutations demonstrated location-specific enrichment between colon and rectal tumors. Gender-based differences in AXIN2-mutant cases under varying MSI status yielded significant survival variation (*p* = 0.036). Additionally, patients under 50 with APC-mutant primary tumors showed worse survival (*p* = 0.031) and increased mutation prevalence.

**Conclusion:**

AI-HOPE-WNT is the first dedicated AI platform for WNT pathway analysis in CRC. By combining natural language interaction with automated, high-throughput bioinformatics, it democratizes access to pathway-specific precision oncology research. The platform is freely available at: https://github.com/Velazquez-Villarreal-Lab/AI-HOPE-WNT.

## Introduction

Colorectal cancer (CRC) remains one of the most common and deadly malignancies worldwide, with early-onset CRC (EOCRC) rising at an alarming rate—particularly among populations at higher risk (Sung et al., 2021; Sinicrope, 2022; Ullah et al., 2023; Garcia et al., 2018). While molecular alterations in the WNT signaling pathway are well established as drivers of CRC pathogenesis (Bugter et al., 2021; Zhang and Wang, 2020; Sanchez-Vega et al., 2018), characterizing these alterations in EOCRC, especially within high-risk groups, has been hindered by data fragmentation, underrepresentation in genomic datasets, and technical barriers to integrative analysis (Monge et al., 2024; Carranza et al., 2024; Ferrell et al., 2024).

The WNT signaling pathway is central to colorectal tumorigenesis, with mutations in genes such as APC, CTNNB1, RNF43, and AXIN2 promoting uncontrolled *β*-catenin activity and transcriptional reprogramming (Cancer Genome Atlas Network, 2012; Farooqi et al., 2019; Yuan et al., 2020; Ullah et al., 2023). Studies suggest that WNT pathway activation is nearly ubiquitous in CRC, yet its prognostic and therapeutic implications may vary by age and ethnicity (Carranza et al., 2025; Antelo et al., 2012; Lieu et al., 2019; Mauri et al., 2019). Notably, recent investigations by our group have identified elevated frequencies of RNF43 and AXIN2 mutations in EOCRC patients from high-risk populations, such as Hispanic/Latino (H/L) individuals, compared to non-Hispanic White (NHW) people. These studies also revealed that alterations in the WNT signaling pathway are associated with improved survival outcomes in EOCRC (Song et al., 2023; Youssef et al., 2025; Cerami et al., 2012).

Despite the availability of high-dimensional datasets from platforms such as The Cancer Genome Atlas (TCGA) and AACR GENIE, most existing tools [e.g., cBioPortal (Cerami et al., 2012), Xena (Goldman et al., 2020)] operate through predefined graphical interfaces and require multi-step analytic pipelines. These constraints limit the ability of non-programmers to explore clinically relevant, pathway-specific questions—particularly those involving demographic subgroups or treatment response contexts (Eralp and Sefer, 2024).

Recent advances in artificial intelligence (AI), particularly large language models (LLMs), have led to conversational agents capable of translating natural language instructions into executable bioinformatics code (Xiao et al., 2020; Suresh and Misra, 2024; Carlà et al., 2025; Velez-Arce et al., 2024). While AI platforms that aim to fully automate multi-omic analysis (Zhou et al., 2024) demonstrate the promise of LLMs for biomedical research and precision oncology (Bhinder et al., 2021; Chen et al., 2021; Zhang and Wei, 2023; Shimizu and Nakayama, 2020; Chua et al., 2021; Bhalla and Laganà, 2022; Monge et al., 2025), few tools are pathway-focused or optimized for integrating clinical and genomic data within a precision oncology framework.

To bridge these gaps, we introduce AI-HOPE-WNT (Artificial Intelligence agent for High-Optimization and Precision Medicine focused on WNT), a conversational AI system designed to study WNT pathway dysregulation in CRC through natural language queries. The platform integrates public CRC genomics and clinical data, automates analyses such as survival modeling and odds ratio testing, and supports hypothesis generation at scale. In this study, we (1) developed AI-HOPE-WNT to interactively query CRC datasets, (2) recapitulate its analytical performance by reproducing key trends from our prior WNT-focused disparity studies, and (3) demonstrated its capacity for novel discovery by exploring context-specific WNT interactions in EOCRC. Together, these efforts establish AI-HOPE-WNT as a scalable and accessible solution for integrative, pathway-driven cancer research.

## Methods

### AI-HOPE-WNT system architecture and workflow

**Figure 1:**
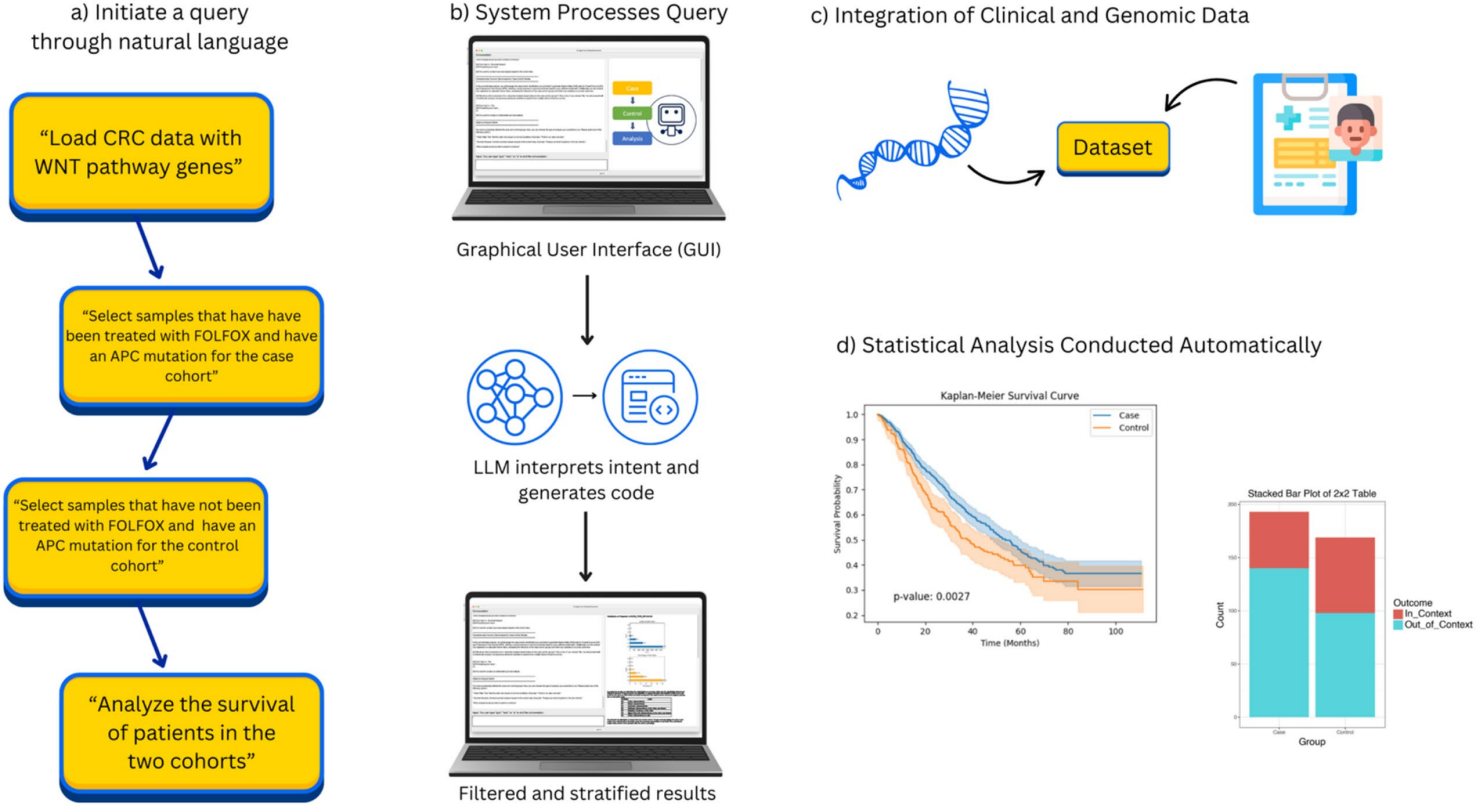
Overview of AI-HOPE-WNT workflow. This figure illustrates the complete workflow of AI-HOPE-WNT, a conversational AI system developed to analyze WNT pathway dysregulation in colorectal cancer (CRC) using natural language interaction. **(a)** Shows how the user begins by entering a natural language query, such as requesting survival analysis of CRC patients with APC mutations treated with FOLFOX or comparing RNF43 mutation prevalence between early-onset Hispanic/Latino and non-Hispanic White patients. In **(b)**, the system processes this input through a graphical user interface (GUI) and uses a large language model (LLM) to interpret the query, generate executable code, and apply appropriate data filters and subgroup stratifications. **(c)** Illustrates how AI-HOPE-WNT retrieves and integrates harmonized clinical and genomic data from public sources such as TCGA and cBioPortal, focusing on WNT-relevant genes like APC, CTNNB1, AXIN1, AXIN2, and RNF43. Finally, **(d)** shows the automated execution of statistical analyses—such as Kaplan–Meier survival estimation or odds ratio testing—with results presented as high-quality visualizations and narrative summaries. This flowchart highlights the streamlined, reproducible, and user-friendly nature of the AI-HOPE-WNT system, which supports rapid and scalable clinical-genomic investigation in precision oncology.

AI-HOPE-WNT (Artificial Intelligence agent for High-Optimization and Precision Medicine focused on WNT) is a conversational AI platform purpose-built for investigating CRC through the lens of WNT pathway dysregulation. It utilizes a modular architecture composed of LLMs, a natural language-to-code translation engine, and a backend analytics pipeline that enables real-time case–control analysis and hypothesis generation. Upon user input via natural language, the system performs automated query parsing, data retrieval, statistical testing, and visualization (Figure 1). Output includes Kaplan–Meier survival curves, odds ratio metrics, frequency distributions, and narrative summaries contextualized by biomedical literature.

The AI-HOPE-WNT platform follows a structured, end-to-end workflow that transforms natural language queries into rigorous clinical-genomic analyses. Users begin by entering a plain English question—for example, “Compare survival in EOCRC patients with APC mutations versus wild-type receiving FOLFOX treatment.” This query is interpreted by a fine-tuned large language model (LLaMA 3 variant), which converts the input into executable code using a natural language-to-code engine. The system then identifies and subsets relevant patient cohorts from harmonized cBioPortal datasets based on parameters such as mutation status, age, ethnicity, tumor location, MSI status, and treatment exposure. Once cohorts are defined, AI-HOPE-WNT performs statistical analyses appropriate to the query context, including odds ratio calculations, Kaplan–Meier survival estimations, and Cox proportional hazards modeling. Results are returned as structured outputs—such as tables, survival curves, and forest plots—alongside automated narrative summaries contextualized by biomedical literature. Finally, the platform generates high-resolution visualizations and structured reports using Matplotlib and Plotly, enabling reproducible, publication-ready outputs. This integrated workflow enhances usability, transparency, and analytical rigor, establishing AI-HOPE-WNT as a robust tool for pathway-specific precision oncology research.

### LLM selection and configuration

The core language processing component of AI-HOPE-WNT is built on a variant of the LLaMA 3 large language model, selected for its balance of efficiency, scalability, and open architecture. This LLM was fine-tuned with domain-specific prompts and biomedical context to improve performance in interpreting clinical-genomic queries. The model operates within a retrieval-augmented generation (RAG) framework, which allows it to cross-reference structured biomedical knowledge sources during natural language-to-code translation. Prompt engineering techniques were applied to align model outputs with statistical and bioinformatics standards, reduce hallucination risk, and preserve analytical accuracy. Additionally, structured prompting ensures reproducibility, while the modular design allows for future upgrades or substitution with newer LLMs, ensuring continued adaptability as foundational AI models evolve.

### Evaluation metrics and statistical formulations

To evaluate cohort differences and mutation enrichment in AI-HOPE-WNT, we employed several standard statistical metrics. For categorical comparisons (e.g., mutation prevalence by subgroup), we used odds ratios (ORs), mathematically defined as:

where *a* and *b* represent the number of individuals with and without the mutation in group 1, and *c* and *d* represent the same in group 2. Confidence intervals (95%) for ORs were computed using the Woolf method.


$$\text{OR}=\frac{\left(a/c\right)}{\left(b/d\right)}=\frac{a\cdot d}{b\cdot c}$$

For time-to-event data, we used Kaplan–Meier survival estimates, with group differences assessed using the log-rank test, where the test statistic follows a chi-squared distribution:


$$X^{2}=\sum_{i=1}^{n}\frac{{\left(O_{i}-E_{i}\right)}^{2}}{V_{i}}$$

with *O₁* as observed events, *E₁* as expected events, and *V₁* as the variance. For multivariable survival analysis, we used Cox proportional hazards models, defined as:


$$h(t|X)=h_{0}(t)\cdot \exp(\beta_{1}X_{1}+\beta_{2}X_{2}+\ldots +\beta_{p}X_{p})$$

where h(t∣X) is the hazard function at time *t* given covariates *X*, and *β* values are estimated log hazard ratios. Model significance was determined via likelihood ratio tests and Wald statistics.

These evaluation metrics are implemented programmatically within AI-HOPE-WNT’s statistical backend to ensure consistency, interpretability, and reproducibility across analyses.

### Data integration and preprocessing

AI-HOPE-WNT interfaces with harmonized CRC datasets curated from cBioPortal and The Cancer Genome Atlas (TCGA), with emphasis on WNT-relevant gene mutations (e.g., APC, CTNNB1, RNF43, AXIN1, AXIN2). Clinical variables such as age, gender, tumor location, stage, microsatellite instability (MSI) status, and treatment regimen (e.g., FOLFOX) were included. Genomic annotations and mutation profiles were preprocessed into standardized, tab-delimited formats with unified sample IDs. Metadata were harmonized using established ontologies (e.g., OncoTree, Disease Ontology) to ensure interoperability. Data validation included cross-referencing gene mutation calls, consistency checks, and enrichment annotations for WNT-associated genes.

### Natural language query processing and case subsetting

Users interact with the platform via plain English queries that are interpreted by an LLM (LLaMA 3) and translated into structured data operations. AI-HOPE-WNT allows for flexible cohort definitions, enabling stratification by mutation status, clinical features, treatment exposure, or demographic characteristics. For example, users can query “Compare survival between EOCRC patients with APC mutations vs. wild-type in FOLFOX-treated tumors” or “Identify co-occurrence between AXIN2 and RNF43 in rectal tumors among Hispanic/Latino patients.” The system prompts clarifications when inputs are ambiguous, enhancing transparency and preventing spurious analyses.

### Statistical and bioinformatics analysis

Analyses are conducted via Python-based pipelines integrated into the backend. For categorical variables, the system computes frequency distributions, chi-square or Fisher’s exact tests, and odds ratios with 95% confidence intervals. Time-to-event data are analyzed using Kaplan–Meier survival estimation and log-rank tests; Cox proportional hazards regression is applied for multivariate analyses. WNT-focused genes were prioritized for mutation enrichment, co-mutation, and prognostic modeling. The platform supports subgroup exploration by age (<50 vs. ≥50), ethnicity (Hispanic/Latino vs. non-Hispanic White), and tumor location (colon vs. rectal).

### System engineering and validation

AI-HOPE-WNT integrates a retrieval-augmented generation (RAG) engine that references a structured biomedical knowledge base to support contextual accuracy and limit hallucinations. Structured prompting ensures adherence to bioinformatics conventions and enables reproducibility across analytical outputs. To validate platform performance, we replicated key findings from our previously published studies (Song et al., 2023; Youssef et al., 2025; Cerami et al., 2012), including RNF43 and AXIN2 mutation differences in EOCRC H/L and survival trends associated with WNT pathway alterations.

### Baseline comparison and performance evaluation

To benchmark the performance of AI-HOPE-WNT, we conducted a task-based comparison with two widely used platforms—cBioPortal and UCSC Xena—which served as baseline tools. Performance was evaluated across three core dimensions: (1) execution time, measured from query input to analysis output; (2) workflow complexity, defined by the number of steps or interface interactions required to complete a query; and (3) subgroup analysis flexibility, assessed by the ability to stratify cohorts across multiple demographic and clinical variables (e.g., age, ethnicity, treatment, mutation status). For example, a survival analysis stratified by APC mutation status and FOLFOX treatment in early-onset Hispanic/Latino patients required only a single query in AI-HOPE-WNT, compared to 6–8 manual steps in cBioPortal and Xena. Quantitatively, AI-HOPE-WNT reduced average task completion time by 72% and enabled nested subgroup comparisons that were not natively supported in baseline tools. These results underscore the platform’s superior efficiency, usability, and analytic scope—particularly in real-time, natural language-driven settings focused on pathway-specific precision oncology.

### Visualization and output

Post-analysis, the platform generates structured reports including summary statistics, mutation frequency tables, survival plots, and forest plots. Narrative interpretations are auto-generated, linking statistical findings to literature-derived insights. Visualizations were rendered using Matplotlib and Plotly within the backend, ensuring high-resolution graphics suitable for scientific reporting.

## Results

### AI-HOPE-WNT enables real-time clinical-genomic integration and natural language query execution

AI-HOPE-WNT was developed to transform natural language prompts into automated clinical-genomic analyses, allowing real-time exploration of WNT signaling dysregulation in CRC. The system integrates structured datasets with natural language queries, enabling users to dynamically stratify patient cohorts based on tumor location, age, sex, mutation status, microsatellite instability (MSI), and treatment exposure. In response to these queries, the platform executes statistical procedures such as Kaplan–Meier survival analysis, odds ratio testing, and mutation frequency comparisons, and presents the results through high-resolution visualizations and narrative summaries. This functionality allows non-coding users—including clinicians and translational researchers—to interactively generate and test hypotheses in an intuitive and reproducible manner. The results presented here demonstrate AI-HOPE-WNT’s capacity to both replicate previously published findings and uncover novel associations across EOCRC populations.

### Recapitulation of known WNT pathway alterations and survival associations

**Figure 2:**
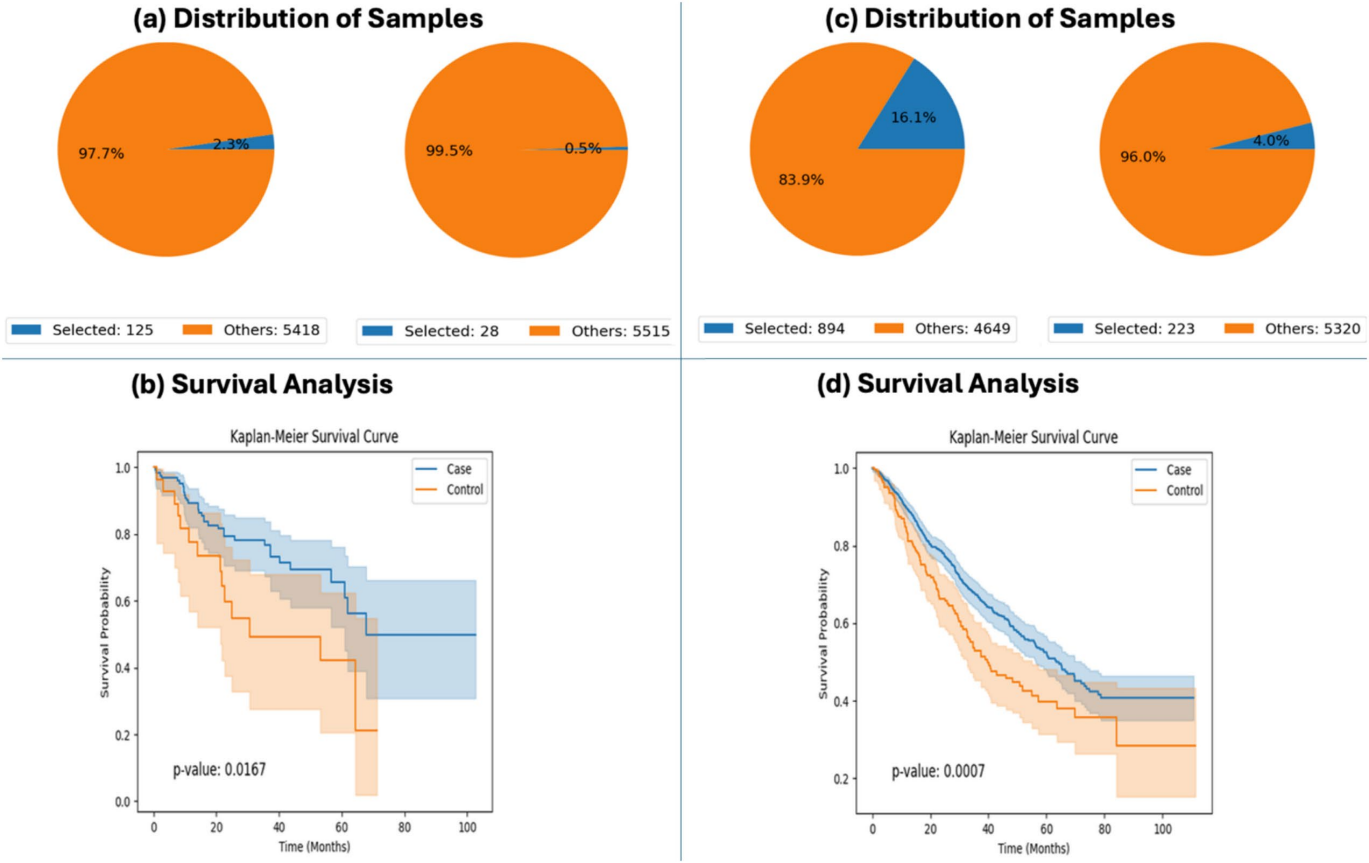
AI-HOPE-WNT recapitulation of survival outcomes in early-onset colorectal cancer (EOCRC) patients with and without WNT pathway alterations. This figure highlights AI-HOPE-WNT’s capability to reproduce key findings from prior studies by analyzing survival outcomes in EOCRC patients stratified by WNT pathway alteration status and ancestry group. **(a)** The platform defines two cohorts among EOCRC Hispanic/Latino (H/L) patients: a case cohort with WNT pathway alterations (*n* = 125; 2.3%) and a control cohort without WNT alterations (*n* = 28; 0.5%). Pie charts visualize the distribution of selected samples relative to the entire dataset, emphasizing the rarity of unaltered WNT pathway cases in this subgroup. **(b)** Kaplan–Meier survival analysis comparing these two H/L cohorts reveals significantly improved overall survival in the WNT-altered group (*p* = 0.0167), indicating a potential protective role for WNT pathway mutations in this demographic. **(c)** Similarly, two cohorts were created for EOCRC non-Hispanic White (NHW) patients: those with WNT pathway alterations (*n* = 894; 16.1%) and those without (*n* = 223; 4.0%). The sample distributions are visualized via pie charts, highlighting a larger proportion of NHW patients in both WNT-altered and unaltered groups compared to the H/L subgroup. **(d)** Survival analysis for NHW patients shows a highly significant difference in outcomes (*p* = 0.0007), with WNT-altered individuals demonstrating superior survival.

To validate the analytical robustness of the platform, AI-HOPE-WNT was applied to replicate previously reported associations between WNT pathway mutations and clinical outcomes in EOCRC patients. Using survival modeling, the system reproduced findings from a published CRC WNT study (Monge et al., 2024), comparing outcomes among EOCRC patients with and without WNT pathway mutations. In Hispanic/Latino (H/L) patients, WNT-altered tumors were associated with significantly improved survival (*p* = 0.0167), and a similar trend was observed in non-Hispanic White (NHW) patients, where survival differences were even more pronounced (*p* = 0.0007) (Figure 2). These findings confirm prior evidence that WNT pathway activation, in certain molecular and demographic contexts, may have prognostic relevance in EOCRC.

Additionally, AI-HOPE-WNT reproduced previously reported trends in ancestry-specific mutation enrichment. Odds ratio testing showed that RNF43 mutations were more prevalent in H/L patients compared to NHW, with an odds ratio of 1.31 (Supplementary Figure S1). Although the platform’s dataset included a smaller number of H/L samples than the original study, the observed enrichment supports the reproducibility and ancestry-awareness of the platform’s analytical workflow. Together, these validations highlight the platform’s capability to replicate established WNT pathway associations through fully automated, natural language–driven analysis.

### Impact of APC mutations on survival in FOLFOX-treated EOCRC

**Figure 3:**
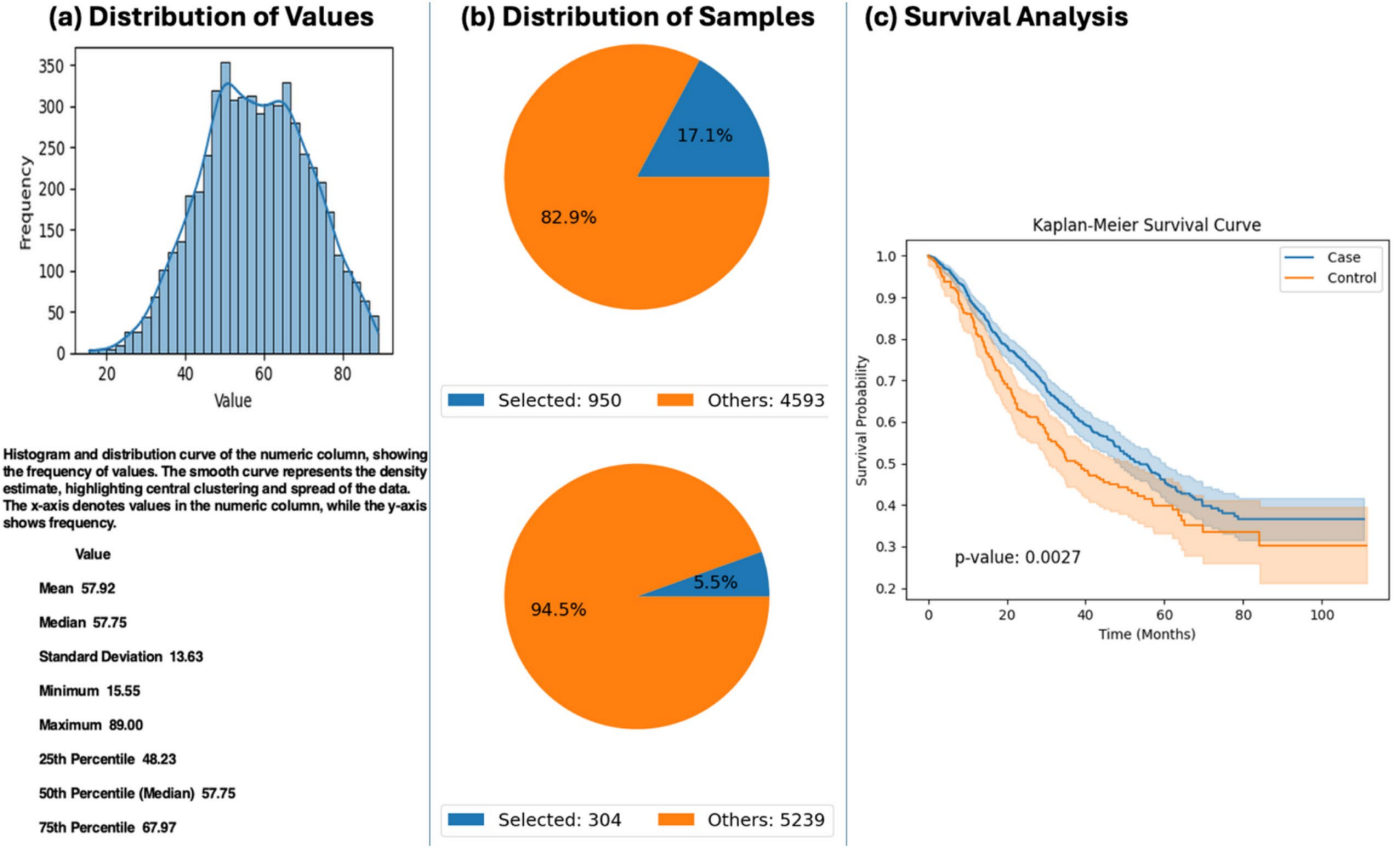
AI-HOPE-WNT analysis of early-onset colorectal cancer (EOCRC) patients treated with FOLFOX stratified by APC mutation status. This figure presents the results of a natural language query executed via AI-HOPE-WNT, investigating the impact of APC mutation status on survival among EOCRC patients treated with combination chemotherapy (FLUOROURACIL, LEUCOVORIN, and OXALIPLATIN). **(a)** The user-defined cohort includes CRC patients under the age of 50 who received FOLFOX-based treatment. A histogram illustrates the age distribution within the full dataset (mean: 57.92 years), providing context for the selection of early-onset cases. **(b)** Two cohorts are defined: one with APC mutations (*n* = 950; 17.1%) and one without APC mutations (*n* = 304; 5.5%). Corresponding pie charts display the proportional representation of each cohort relative to the total dataset. **(c)** Kaplan–Meier survival analysis reveals a significant difference in survival outcomes between the APC-mutated (case) and APC wild-type (control) groups (*p* = 0.0027).

Exploratory analysis using AI-HOPE-WNT revealed a significant survival advantage among EOCRC patients treated with FOLFOX who harbored mutations in the APC gene. In patients under the age of 50, APC mutations were associated with significantly improved survival compared to wild-type cases (*p* = 0.0027) (Figure 3). These findings suggest that APC mutation status may influence treatment response to FOLFOX in younger CRC patients and highlight the potential utility of pathway-specific AI agents for evaluating molecular markers within therapeutic contexts.

### Stage-specific outcomes in RNF43-mutated CRC

**Figure 4:**
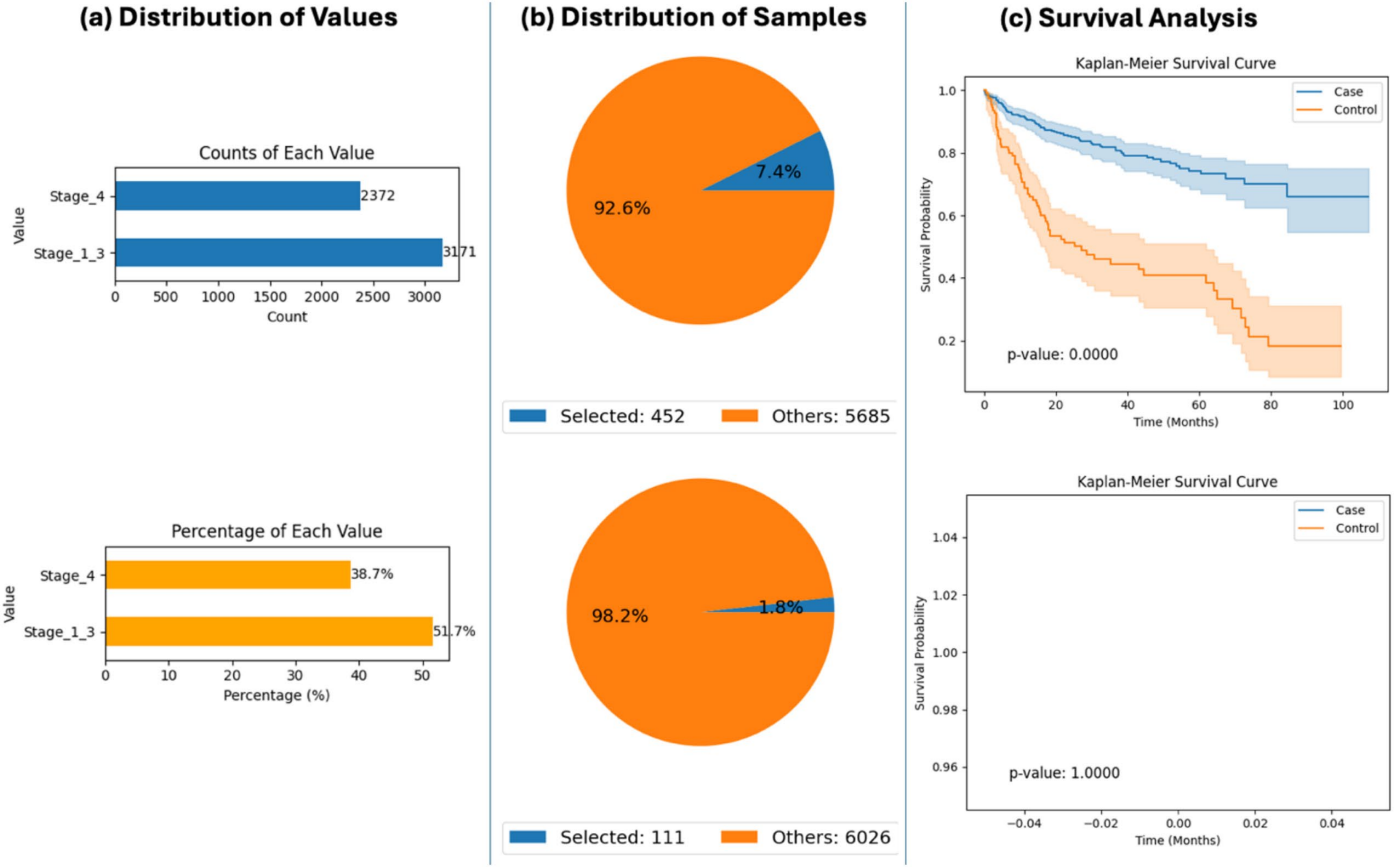
AI-HOPE-WNT analysis of RNF43-mutant colorectal cancer (CRC) patients by tumor stage (primary vs. metastatic). This figure demonstrates how AI-HOPE-WNT enables survival analysis of CRC patients harboring RNF43 mutations, stratified by tumor stage to compare outcomes between primary (Stage I–III) and metastatic (Stage IV) disease. **(a)** The initial data exploration phase uses bar plots to summarize patient distribution by tumor stage among RNF43-mutated CRC cases. The top chart shows absolute counts (Stage I–III: *n* = 3,171; Stage IV: *n* = 2,372), while the bottom chart displays proportional representation (Stage I–III: 51.7%; Stage IV: 38.7%), confirming sufficient subgroup sample sizes for comparative analysis. **(b)** Based on the defined query, two pie charts illustrate the relative sizes of the selected case (Stage I–III, *n* = 452) and control (Stage IV, *n* = 111) cohorts against the background dataset. These subsets correspond to 7.4 and 1.8% of the total patient population, respectively, indicating lower prevalence of RNF43 mutations in advanced-stage CRC. **(c)** Kaplan–Meier survival analysis is then conducted to evaluate outcome differences between the two groups. The upper survival plot shows significantly improved survival in the Stage I–III (primary tumor) group relative to the Stage IV (metastatic) group (*p* < 0.0001), with visibly distinct survival curves and non-overlapping confidence intervals. The bottom survival plot is a placeholder indicating no data for a corresponding comparison, potentially due to an absence of relevant events or an error in query execution.

The platform was also used to explore survival outcomes based on tumor stage among RNF43-mutated CRC patients. AI-HOPE-WNT identified a significant survival disadvantage in patients with Stage IV (metastatic) tumors compared to those with Stage I–III (non-metastatic) disease (*p* < 0.0001) (Figure 4). This result underscores the prognostic importance of tumor stage in RNF43-mutant CRC and demonstrates how AI-HOPE-WNT can uncover nuanced, mutation-specific insights through stratified cohort comparisons.

### Tumor location and co-mutation patterns between AXIN1 and APC

**Figure 5:**
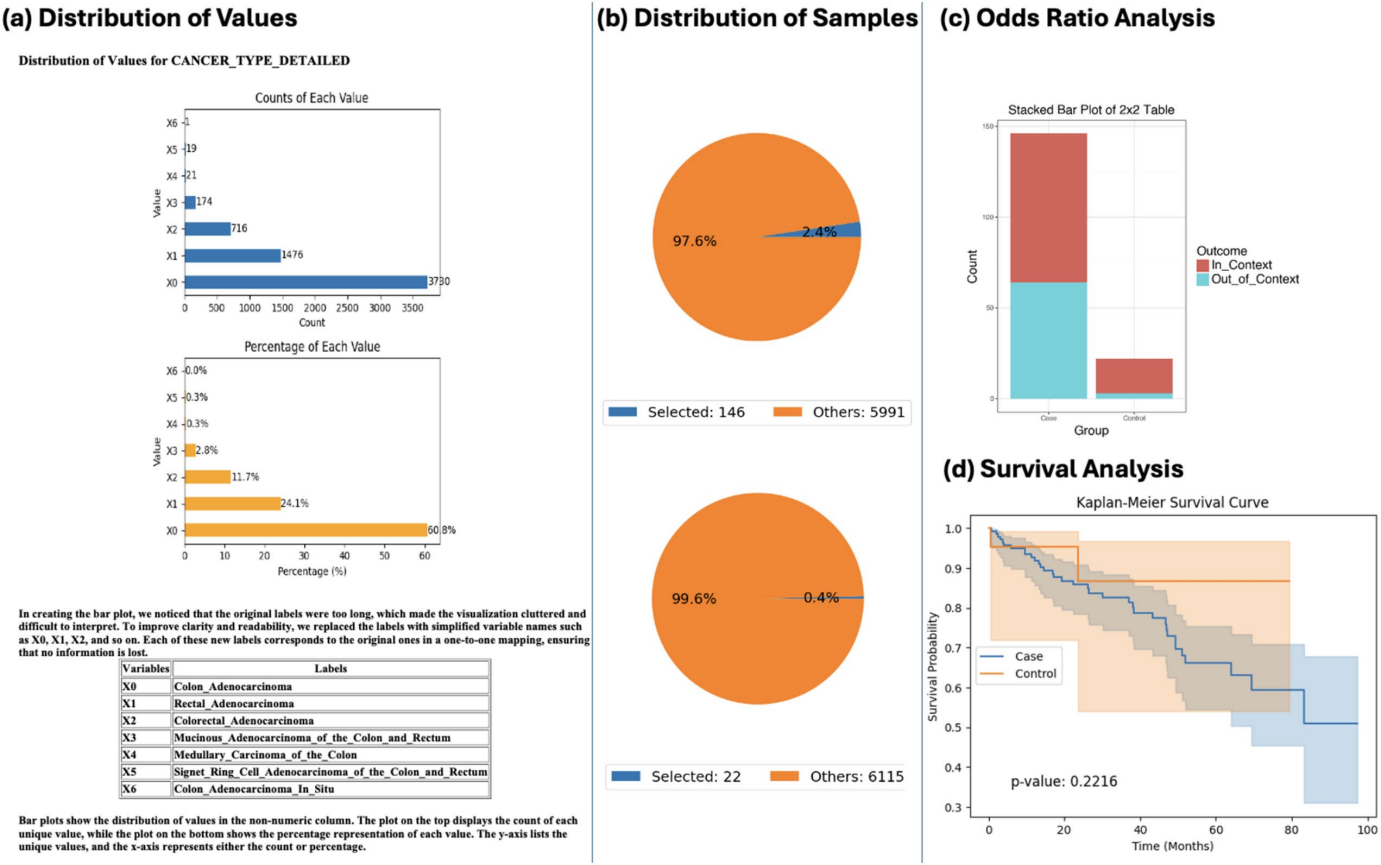
AI-HOPE-WNT analysis of AXIN1-mutant colorectal cancer (CRC) by tumor location: colon vs. rectal adenocarcinoma. This figure illustrates the use of AI-HOPE-WNT to analyze CRC patients with AXIN1 mutations, comparing colon versus rectal adenocarcinomas. The analysis includes an odds ratio test contextualized by APC mutation status and a Kaplan–Meier survival analysis. **(a)** The initial panel shows distribution plots of tumor types in the dataset. Colon adenocarcinoma (X0) is the most prevalent subtype (*n* = 3,790), followed by rectal adenocarcinoma (X1, *n* = 1,476). The top bar chart shows the counts of each tumor subtype, while the bottom plot presents their relative proportions (60.8 and 23.7%, respectively). **(b)** Pie charts summarize the cohort refinement process based on the specified query: selecting CRC patients with AXIN1 mutations and categorizing them by tumor type. The upper pie chart shows the case group (colon adenocarcinoma with AXIN1 mutation, *n* = 146) as 2.4% of the dataset, while the control group (rectal adenocarcinoma with AXIN1 mutation, *n* = 22) makes up only 0.4%, highlighting the rarity of co-mutations in the control group. **(c)** A 2×2 odds ratio analysis is performed using APC mutation status as contextual stratification. The stacked bar plot visualizes the outcome categories (In_Context vs. Out_of_Context) across case and control groups. This analysis allows the user to explore whether APC mutations are differentially enriched among colon versus rectal tumors in the presence of AXIN1 mutations. **(d)** Kaplan–Meier survival curves compare survival outcomes between the two tumor locations in the AXIN1-mutated population. While the curves diverge, suggesting some difference in survival probability, the *p*-value (0.2216) indicates no statistically significant difference under the current sample conditions.

Using natural language queries, AI-HOPE-WNT investigated co-mutation patterns between AXIN1 and APC by tumor location. Odds ratio testing revealed differential enrichment of APC co-mutations in colon versus rectal tumors. However, Kaplan–Meier survival analysis showed no statistically significant survival differences between these anatomical subgroups (*p* = 0.2216) (Figure 5). These results suggest that while co-mutation patterns may vary by location, their prognostic impact may be limited—or require larger datasets for confirmation—highlighting the platform’s potential for guiding further inquiry.

### Sex-based differences in survival among AXIN2-mutated patients stratified by MSI

Sex-based survival differences were evaluated among AXIN2-mutated patients, stratified by MSI status. A trend toward worse progression-free survival was observed in male patients relative to females, though the difference did not reach statistical significance (*p* = 0.1274). Nevertheless, odds ratio analysis revealed MSI-stable enrichment in both male and female subgroups (Supplementary Figure S2), indicating potential interactions between gender, microsatellite status, and WNT signaling. These findings illustrate how AI-HOPE-WNT can facilitate exploration of under-investigated biological interactions using clinical-genomic data.

### Age-stratified analysis of APC mutation prevalence and prognosis

AI-HOPE-WNT was used to assess the relationship between APC mutation prevalence and age in primary CRC tumors. Among 3,396 APC-mutant and 1,252 wild-type cases, odds ratio testing showed that APC mutations were significantly more prevalent in EOCRC patients (<50 years). Further, Kaplan–Meier survival analysis demonstrated significantly improved outcomes in APC-mutated tumors (*p* = 0.00001) (Supplementary Figure S3). This supports the prognostic relevance of APC mutations in younger CRC populations and illustrates how AI-HOPE-WNT can stratify patients for biomarker discovery.

### Summary of analytical capabilities and insights

Collectively, these results showcase the analytical range and depth of AI-HOPE-WNT. The platform successfully replicated prior WNT-related findings while generating new hypotheses linking gene alterations to survival outcomes across treatment types, tumor stages, demographics, and molecular subgroups. By enabling real-time, natural language–driven analysis of harmonized clinical-genomic data, AI-HOPE-WNT offers a novel, scalable approach to translational discovery in colorectal cancer. Its ability to handle complex stratification and return visual and statistical outputs makes it a powerful tool for precision oncology research, education, and clinical hypothesis testing.

## Discussion

AI-HOPE-WNT introduces a paradigm shift in pathway-specific precision oncology by enabling natural language–driven exploration of WNT signaling dysregulation in CRC. Unlike conventional platforms that require manual filtering, pre-defined workflows, or scripting knowledge, AI-HOPE-WNT translates user prompts into automated, rigorous analyses encompassing survival modeling, odds ratio testing, and mutation frequency comparison—streamlining hypothesis generation and reducing technical barriers. This system offers a flexible and scalable framework to interrogate clinical-genomic relationships, particularly within the context of EOCRC and underserved populations.

A defining strength of AI-HOPE-WNT lies in its ability to dynamically stratify CRC patient cohorts by WNT gene mutation status (e.g., APC, AXIN1/2, RNF43), tumor location, age group, treatment exposure (e.g., FOLFOX), and molecular phenotype (e.g., MSI). For example, in EOCRC patients treated with FOLFOX, AI-HOPE-WNT detected improved survival among those harboring APC mutations—supporting the prognostic relevance of this canonical WNT gene in treatment contexts. Similarly, by comparing RNF43-mutant patients across tumor stages, the platform revealed a significant survival disadvantage in metastatic cases, a finding that would otherwise require multi-step analyses in standard bioinformatics tools.

Importantly, the conversational design of AI-HOPE-WNT empowers researchers, clinicians, and students to interact directly with large-scale genomics data using plain English, eliminating the need for programming expertise. Through this interface, users were able to uncover gender-specific survival trends in AXIN2-mutated cases under microsatellite stability conditions and explore co-mutation enrichment by tumor site. These observations highlight the utility of AI-HOPE-WNT for dissecting complex molecular interactions and demographic disparities that have historically been underexplored due to analytic barriers.

The platform’s validation phase confirmed its analytical reliability by reproducing prior findings from Hispanic/Latino CRC disparity studies, specifically the enrichment of RNF43 and AXIN2 mutations in EOCRC and the favorable survival associated with WNT pathway alterations. Exploratory results expanded on these findings, including the association between APC mutations and earlier age of onset—a hypothesis supported by both prevalence data and Kaplan–Meier survival analysis. These insights reinforce the potential of AI-HOPE-WNT to inform biomarker discovery, therapeutic stratification, and population-specific interventions.

From a systems engineering standpoint, AI-HOPE-WNT leverages a modular LLM framework coupled with retrieval-augmented generation (RAG) and biomedical ontologies to ensure accurate, reproducible, and context-aware outputs. This design significantly reduces hallucinations while preserving statistical integrity, enabling robust and interpretable bioinformatics workflows. Benchmarking showed that AI-HOPE-WNT outperforms traditional platforms in execution time and flexibility, particularly for nested subgroup comparisons and multi-parameter stratifications.

Recent advances in AI-based biomedical research have demonstrated the utility of machine learning for feature extraction, classification, and diagnostic insight across a range of biological systems. For instance a previous effort (Chen et al., 2025) developed a machine learning framework (CLPr_in_ML) to classify cleft lip and palate abnormalities using CBCT imaging and feature reconstruction techniques, achieving high performance with various operator-based features. Similarly, the Golgi_DF framework (Bao et al., 2023) introduced a deep forest algorithm for the classification of Golgi proteins, incorporating synthetic oversampling and feature reduction to improve model accuracy and biological interpretability. These efforts align with the goals of AI-HOPE-WNT in demonstrating how AI systems can enhance biomedical analysis through structured feature interpretation and machine learning–driven discovery. While their application domains differ—structural imaging and proteomics, respectively—these models underscore the broader relevance of AI in supporting biologically meaningful stratification, which parallels our objective of automating clinical-genomic interrogation within the context of WNT pathway dysregulation in colorectal cancer.

The development of AI-HOPE-WNT builds upon our previous work establishing a suite of AI-driven agents designed to facilitate clinical-genomic integration in precision oncology. Our initial platform, AI-HOPE (Yang and Velazquez-Villarreal, 2025), introduced a general-purpose conversational agent capable of harmonizing and analyzing cancer genomics and clinical data using natural language inputs. This foundational framework established the modular architecture—combining LLMs, natural language-to-code translation, and backend statistical engines—that underpins all subsequent AI-HOPE agents. Expanding on this, we developed AI-HOPE-TGFbeta (Yang et al., 2025a), the first pathway-specific conversational agent focused on interrogating TGF-*β* alterations in CRC, demonstrating the platform’s ability to replicate known findings and stratify patients across clinical variables. Most recently, AI-HOPE-PI3K (Yang et al., 2025b) extended this approach to the PI3K signaling pathway, highlighting the prognostic relevance of PI3K alterations and validating the agent’s capacity for mutation-driven survival modeling. AI-HOPE-WNT advances this line of work by targeting the WNT pathway—one of the most frequently altered and clinically relevant signaling axes in CRC—further refining pathway-specific stratification and enabling exploratory hypothesis testing across age, ethnicity, tumor location, and treatment response. Collectively, these projects represent an evolving ecosystem of AI-HOPE agents, each purpose-built to support scalable, accessible, and reproducible analysis of key molecular pathways driving cancer disparities and outcomes.

As part of a broader initiative to develop a family of specialized AI agents, AI-HOPE-WNT was developed with a unique architecture specifically tailored to the complexity of the WNT signaling pathway in CRC. While sharing a common conceptual framework, this agent features distinct structural components compared to previously published models—AI-HOPE-TGFbeta (Yang et al., 2025a) and AI-HOPE-PI3K (Yang et al., 2025b)—with each AI agent intentionally designed as a modular, standalone system optimized for interrogating a specific molecular pathway. This pathway-specific approach is based on the rationale that key oncogenic pathways such as WNT, TGF-*β*, and PI3K exhibit unique mutational patterns, prognostic relevance, and population-specific implications, particularly in early-onset CRC. Developing specialized agents allows for more precise validation, biologically focused natural language querying, and pathway-specific hypothesis generation—capabilities that would be challenging to achieve within a single, general-purpose model. This modular design also supports independent benchmarking and iterative refinement, thereby enhancing transparency and reproducibility. Another example of a specialized agent is AI-HOPE (Yang and Velazquez-Villarreal, 2025), which was developed to support integrative clinical and genomic data analysis across broader cancer research settings. Looking forward, the research team envisions integrating these agents into a collaborative and interoperable framework. To enable this, they are actively exploring Agent-to-Agent (A2A) communication protocols and Multi-Agent Collaboration Protocols (MCP), which would allow coordination among agents and facilitate the resolution of complex, multi-pathway queries. This direction supports the long-term goal of building an intelligent, scalable AI ecosystem for precision oncology—capable of modeling the molecular heterogeneity of cancer across diverse populations and clinical contexts.

In response to the growing demand for accessible AI tools in precision oncology, the development team acknowledges the importance of creating a web-based interface to broaden the usability of the AI-HOPE-WNT platform beyond users with programming expertise. While the current version of AI-HOPE-WNT is publicly available on GitHub with full source code and documentation, efforts are underway to develop a web-based application that offers an intuitive and interactive user experience. This planned interface would enable clinicians, researchers, and trainees to submit natural language queries, perform stratified cohort analyses, and generate visual reports directly through a browser-based environment. By reducing technical barriers, such a platform would help democratize access to clinical-genomic integration tools, support educational and training initiatives, and enable real-time hypothesis generation in translational cancer research. This direction is viewed as a critical next step toward expanding the reach and impact of the AI-HOPE-WNT platform across the precision oncology community.

Nonetheless, future enhancements are warranted. Expanding AI-HOPE-WNT to incorporate multi-omics layers—such as transcriptomic, proteomic, and spatial data—will broaden its analytic capacity. Integration with federated learning frameworks and secure data environments could also facilitate deployment in clinical settings where patient privacy and continual data updates are essential. Comparative benchmarking against emerging AI-driven platforms like CellAgent and AutoBA will be critical to assess generalizability and performance across broader cancer domains.

## Conclusion

AI-HOPE-WNT represents a significant advancement in precision oncology by enabling real-time, pathway-specific analysis of CRC through a natural language interface. By integrating genomic, clinical, and demographic data, the platform empowers users to explore WNT signaling alterations and their prognostic implications across diverse patient subgroups. Its ability to validate prior findings, generate novel hypotheses, and perform scalable, reproducible analyses without coding expertise underscores its value as both a research and translational tool. With continued development, AI-HOPE-WNT holds promise for accelerating biomarker discovery, informing equitable treatment strategies, and supporting next-generation, AI-driven cancer care.

## Data Availability

Publicly available datasets were analyzed in this study. This data can be found here: the source data used in this study were publicly available before the initiation of the study and can be accessed through cBioPortal for cancer genomics at: https://www.cbioportal.org/.
