# Supplementary material for: Conversational AI agent for precision oncology: AI-HOPE-WNT integrates clinical and genomic data to investigate WNT pathway dysregulation in colorectal cancer
Source: Front Artif Intell. 2025 Aug 11;8:1624797. doi: 10.3389/frai.2025.1624797 (PMC12375649; doi:10.3389/frai.2025.1624797)
Supplement: Supplementary file 1 [file Data_Sheet_1.docx]

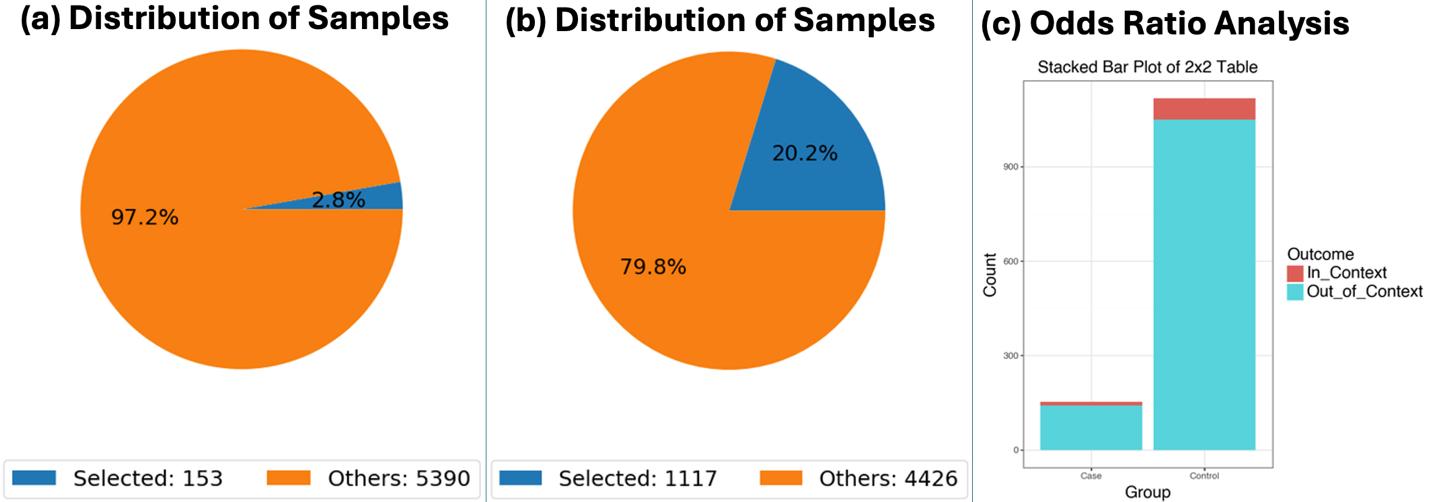


**Figure S1. AI-HOPE-WNT Recapitulation of RNF43 Mutation Frequency in Early-Onset Colorectal Cancer (EOCRC) Patients by Ethnicity**. This figure demonstrates AI-HOPE-WNT’s ability to recapitulate previously reported trends in RNF43 mutation frequency among EOCRC patients, stratified by ethnicity. Specifically, the platform was used to compare RNF43 mutation prevalence between EOCRC Hispanic/Latino (H/L) and non-Hispanic White (NHW) patients using an odds ratio framework. (a) The case cohort includes 153 EOCRC H/L patients under the age of 50 (2.8% of the dataset), identified based on ethnicity filters. A pie chart shows the proportion of selected H/L cases among the total sample population. (b) The control cohort consists of 1,117 EOCRC NHW patients under age 50 (20.2% of the dataset), filtered by race and ethnicity. The pie chart reflects the relative representation of this control group. (c) An odds ratio test evaluates the frequency of RNF43 mutations between the case and control cohorts. The bar plot displays a 2x2 comparison of in-context (RNF43-mutated) and out-of-context (RNF43 wild-type) samples in each group. RNF43 mutations were present in 7.84% of EOCRC H/L samples and 6.09% of EOCRC NHW samples. The resulting odds ratio was 1.313 (95% CI: 0.693–2.486, p = 0.509), indicating a non-significant trend toward higher mutation frequency in the H/L population. This analysis confirms a directional but statistically inconclusive difference in RNF43 mutation rates across ethnic subgroups, consistent with prior observations, and highlights AI-HOPE-WNT’s capacity to perform ethnicity-aware, gene-specific comparisons through natural language–driven querying.


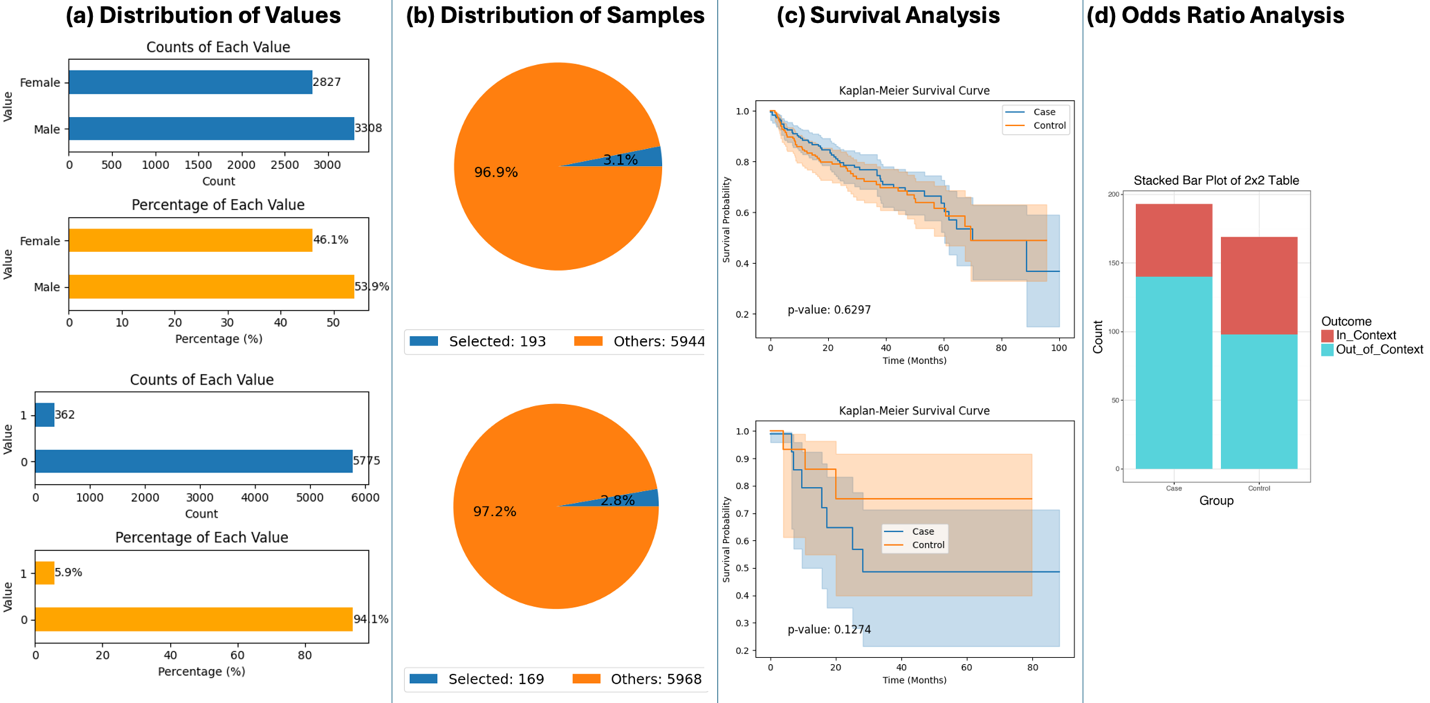


**Figure S2. AI-HOPE-WNT Analysis of Gender-Specific Outcomes in AXIN2-Mutant colorectal cancer (CRC) with microsatellite instability (MSI) Stability Context.** This figure demonstrates AI-HOPE-WNT’s capacity to analyze sex-based differences among CRC patients harboring AXIN2 mutations, integrating MSI status as contextual information for survival and odds ratio analyses. (a) The top bar plots show sex distribution across the dataset, with males (n = 3,308; 53.9%) slightly outnumbering females (n = 2,827; 46.1%). The lower panel shows the distribution of MSI status, where most samples exhibit microsatellite stability (MSS, n = 5,775; 94.1%). (b) Based on the query, AI-HOPE-WNT selects CRC cases with AXIN2 mutations, stratifying them by sex. The pie charts indicate that 193 female cases (3.1%) and 169 male cases (2.8%) met the selection criteria, visualized against the full dataset. (c) Kaplan-Meier survival analysis is conducted for both male and female cohorts. The top curve compares overall survival between AXIN2-mutant females and males, revealing no statistically significant difference (p = 0.6297). The bottom curve evaluates progression-free survival, also showing a non-significant trend (p = 0.1274), though with noticeable divergence in survival probabilities. (d) An odds ratio analysis incorporates MSI status as contextual information. The stacked bar plot contrasts the number of samples within the case and control groups based on MSI stability.


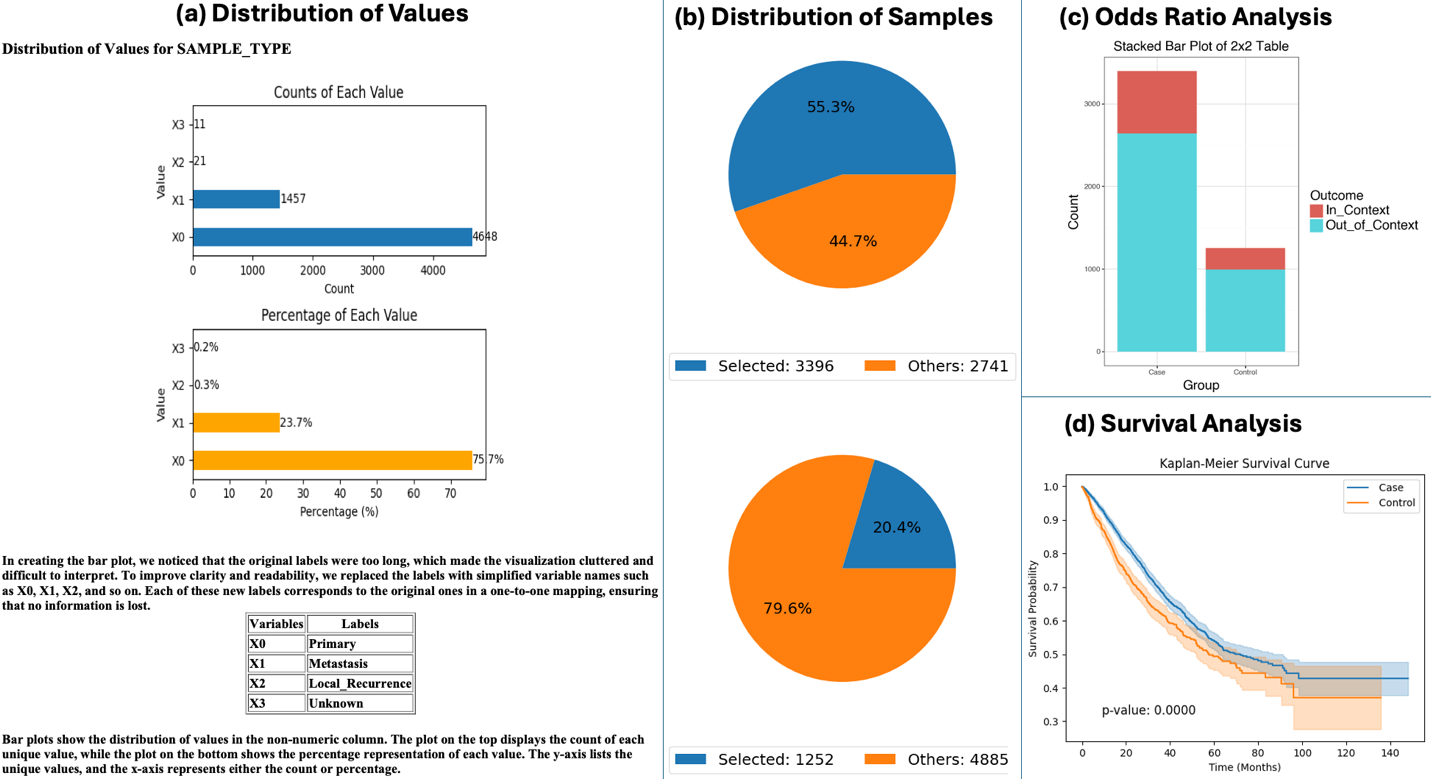


**Figure S3. AI-HOPE-WNT Analysis of Primary colorectal cancer (CRC) Tumors With and Without APC Mutations in the Context of Early-Onset Age (<50 Years)**. This figure illustrates the workflow and outputs generated by AI-HOPE-WNT when comparing primary CRC tumors with and without APC mutations, specifically in the context of patient age being less than 50 years old. (a) The user begins by selecting samples categorized as *Primary* tumors within the CRC dataset. The bar charts depict the distribution of tumor sample types. The majority (75.7%) are classified as primary tumors (X0), followed by metastatic and other less frequent classifications. This step ensures a focused analysis on the primary tumor subset. (b) Two cohorts are created based on APC mutation status. The *case* cohort includes 3,396 APC-mutant primary tumor samples (55.3%), while the *control* cohort includes 1,252 APC wild-type primary tumors (20.4%). Pie charts visualize the proportion of selected samples relative to the dataset, illustrating the subset used in downstream analyses. (c) An odds ratio test is conducted to assess the enrichment of younger patients (age <50) across the two cohorts. The stacked bar chart shows the number of in-context (early-onset) and out-of-context (age ≥50) samples for both groups. A higher number of early-onset patients is observed among APC-mutated tumors, suggesting a possible association between APC alterations and age of onset. (d) A Kaplan-Meier survival analysis is performed to compare overall survival between the APC-mutant and wild-type cohorts within the primary tumor subgroup. The results show a statistically significant difference (p = 0.0000), with patients harboring APC mutations exhibiting improved survival outcomes over time. Confidence intervals are shaded, highlighting the robustness of this survival trend.
